# Progress and current trends in prediction models for the occurrence and prognosis of cancer and cancer-related complications: a bibliometric and visualization analysis

**DOI:** 10.3389/fonc.2025.1556521

**Published:** 2025-07-08

**Authors:** Siyu Li, Wenrui Li, Xiaoxiao Wang, Wanyi Chen

**Affiliations:** Department of Pharmacy, Chongqing University Cancer Hospital, Chongqing, China

**Keywords:** cancer, prediction models, machine learning, bibliometrics, visualization analysis, hotspots and trends

## Abstract

**Objective:**

Prediction models, which estimate disease or outcome probabilities, are widely used in cancer research. This study aims to identify hotspots and future directions of cancer-related prediction models using bibliometrics.

**Methods:**

A comprehensive literature search was conducted in the Science Citation Index Expanded (SCIE) from the Web of Science Core Collection (WoSCC) up to November 15, 2024, focusing on cancer-related prediction models research. Co-occurrence analyses of countries, institutions, authors, journals, and keywords were conducted using VOSviewer 1.6.20. Additionally, keyword clustering, timeline visualization, and burst term analysis were performed with CiteSpace 6.3.

**Results:**

A total of 1,661 records were retrieved from the SCIE. After deduplication and eligibility screening, 1,556 publications were included in the analysis. The bibliometric analysis revealed a consistent annual increase in cancer-related prediction model research, with China and the United States emerging as the leading contributors. The United States, England, and the Netherlands had the strongest collaborative networks. The most frequent keywords, excluding “prediction model” and “predictive model”, included nomogram (frequency=192), survival (191), risk (121), prognosis (112), breast cancer (103), carcinoma (93), validation (87), surgery (85), diagnosis (83), chemotherapy (80), and machine learning (77). Besides, the timeline view analysis indicated that the “#7 machine learning” cluster was experiencing vigorous growth.

**Conclusion:**

Cancer-related prediction models are rapidly advancing, especially in prognostic models. Emerging modeling techniques, such as neural networks and deep learning algorithms, are likely to play a pivotal role in current and future cancer-related prediction model research. Systematic reviews of cancer-related predictive models, which could help clinicians select the optimal model for specific clinical conditions may emerge as potential research directions in this field.

## 1 Introduction

Cancer remains a paramount concern in global public health, imposing a significant burden on both healthcare systems and society due to its rising incidence and mortality rates (1–3). According to statistics from the International Agency for Research on Cancer (IARC), the number of new cancer cases worldwide has surged from 14.1 million in 2012 to nearly 19.98 million in 2022, with corresponding fatalities increasing to 9.74 million (4). The investigation into the etiology, progression, and prognosis of cancer, a complex condition posing a grave threat to human health, has remained a central and challenging area of medical research (5, 6).

The emergence and advancement of bioinformatics, big data analytics, and machine learning have led to the extensive study and application of clinical prediction models (CPMs) in cancer. These models offer novel opportunities for early detection, risk assessment, personalized therapy, and prognostic management of cancer (7–9). CPMs in cancer are generally classified into two main types: cancer incidence prediction models (10–12) and cancer prognosis prediction models (13–15). The former is designed to pinpoint populations at high risk for proactive intervention, while the latter concentrates on predicting post-diagnostic disease progression, recurrence risk, risk of cancer-related complications, and survival probabilities, thereby guiding treatment planning strategies.

Despite the proliferation of studies on cancer-related prediction models, comprehensive reviews and analyses of research trends, technical methodologies, international collaboration networks, and academic influence in this field remain lacking. This study utilizes bibliometric techniques to conduct an extensive review and in-depth analysis of the publications on cancer-related prediction models, providing a thorough synopsis of cancer prediction modeling research. To assist researchers in keeping pace with the latest developments in the field, this study delineates the research momentum, development trajectories, collaborative networks, and the distribution of key authors and institutions, while highlighting key areas of interest and potential future directions.

## 2 Methods

### 2.1 Eligibility criteria

The inclusion criteria for publications were as follows: (1) the publications pertained to cancer-related prediction models; (2) the publications were published in English; (3) the publication date ranged from the inception of the database up to November 15, 2024. The following were excluded: (1) reviews; (2) editorial material; (3) letters, replies, and corrections; (4) duplicate publications; (5) retracted publications; (6) news items.

### 2.2 Search strategy

The primary database for our literature search was the Science Citation Index Expanded (SCIE) of the Web of Science Core Collection (WoSCC). The search was conducted using the following strategy: (“neoplasm” OR “tumor” OR “cancer” OR “oncology” [Title]) AND (“predictive model” OR “prediction model” OR “forecasting model” [Title]).

### 2.3 Bibliometric and visualization analysis

Our study used VOSviewer 1.6.20 to perform co-occurrence analysis on countries, institutions, authors, journals, and keywords within the included publications. Keyword clustering, timeline view, and burst analysis were conducted by CiteSpace 6.3. CiteSpace enables the generation of timeline views and burst term emergence maps across time slices, thereby delineating the evolutionary trajectory of a research field and the historical context of publications within clusters (16). This facilitates an elucidation of the development process, research hotspots, and trends within the field. In contrast, VOSviewer emphasizes the graphical representation of bibliometric data, offering a diverse array of visualizations for areas including keywords, institutions, and authors (17). The integration of these two tools results in a comprehensive and multidimensional analysis, thoroughly uncovering the current state and future trajectory of research in cancer-related prediction models.

**Figure 1:**
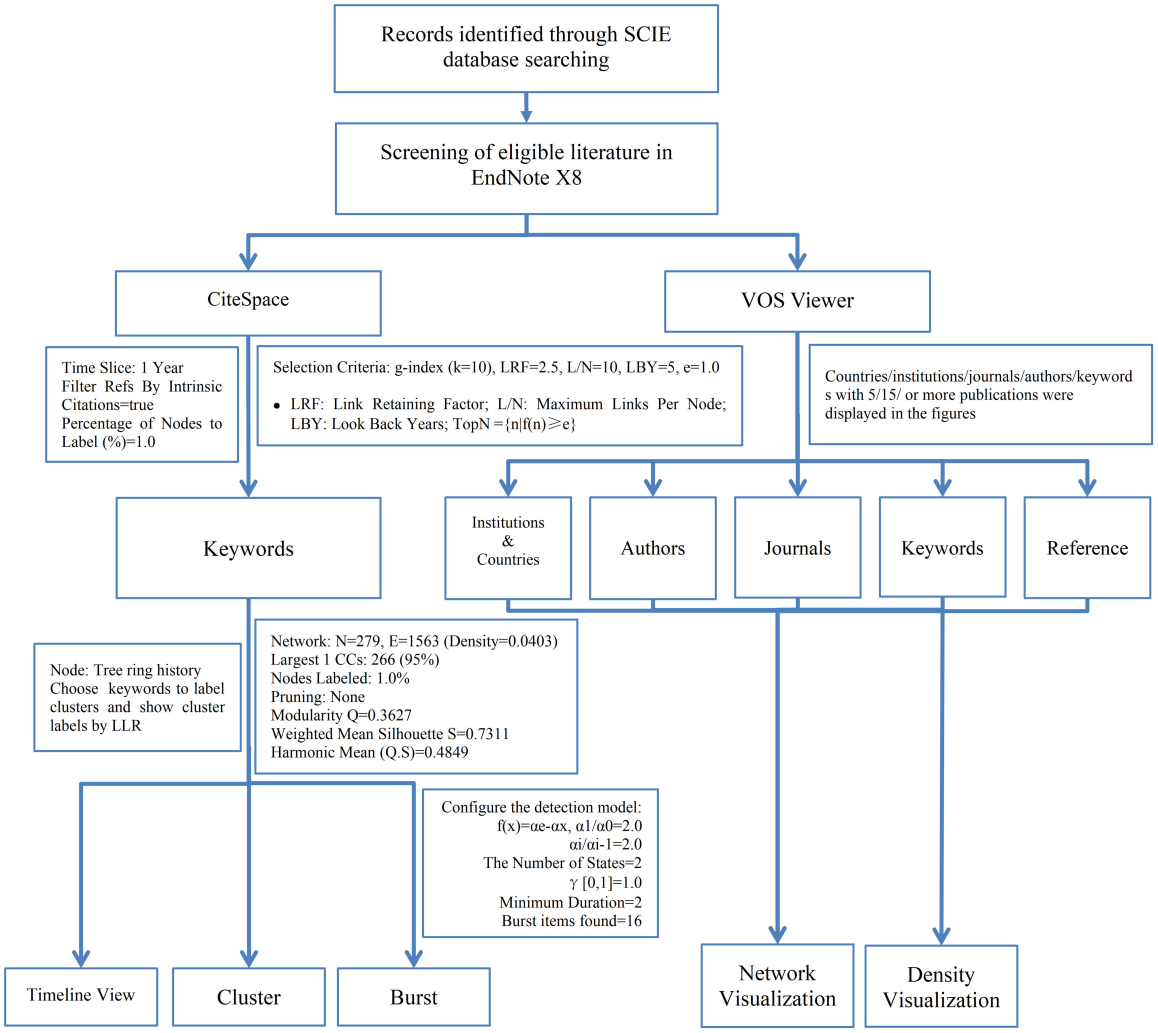
The process of bibliometric analysis.

Publication deduplication and screening were carried out using EndNote X8. The records that met the eligibility criteria were subsequently imported into both CiteSpace 6.3 and VOSviewer 1.6.20 in plain text format. In CiteSpace, the time slice unit was set to one year, and to ensure the aesthetic and readability of the Timeline view, only keywords with a frequency of 20 or higher were displayed (**Figure 1**).

In analyzing authors, Price’s Law (18) and Lotka’s Law (19) were applied to estimate the minimum number of publications for core authors within the field. This established the threshold for author analysis, thereby identifying representative scholars and the core research strengths within the field. ( $M_{\text{min}}=0.749\times \sqrt{N_{max}}$ , where *M*_min_ denotes the minimum number of publications for core authors, and *N*_max_ represents the number of publications by the most productive author.) Additionally, Bradford’s Law (20) was utilized as a bibliometric indicator for identifying core journals. This law reveals the distribution of scientific literature within specific disciplines and facilitates the identification of the most prominently published and influential journals within a specific scientific domain.

## 3 Results

### 3.1 Literature screening

**Figure 2:**
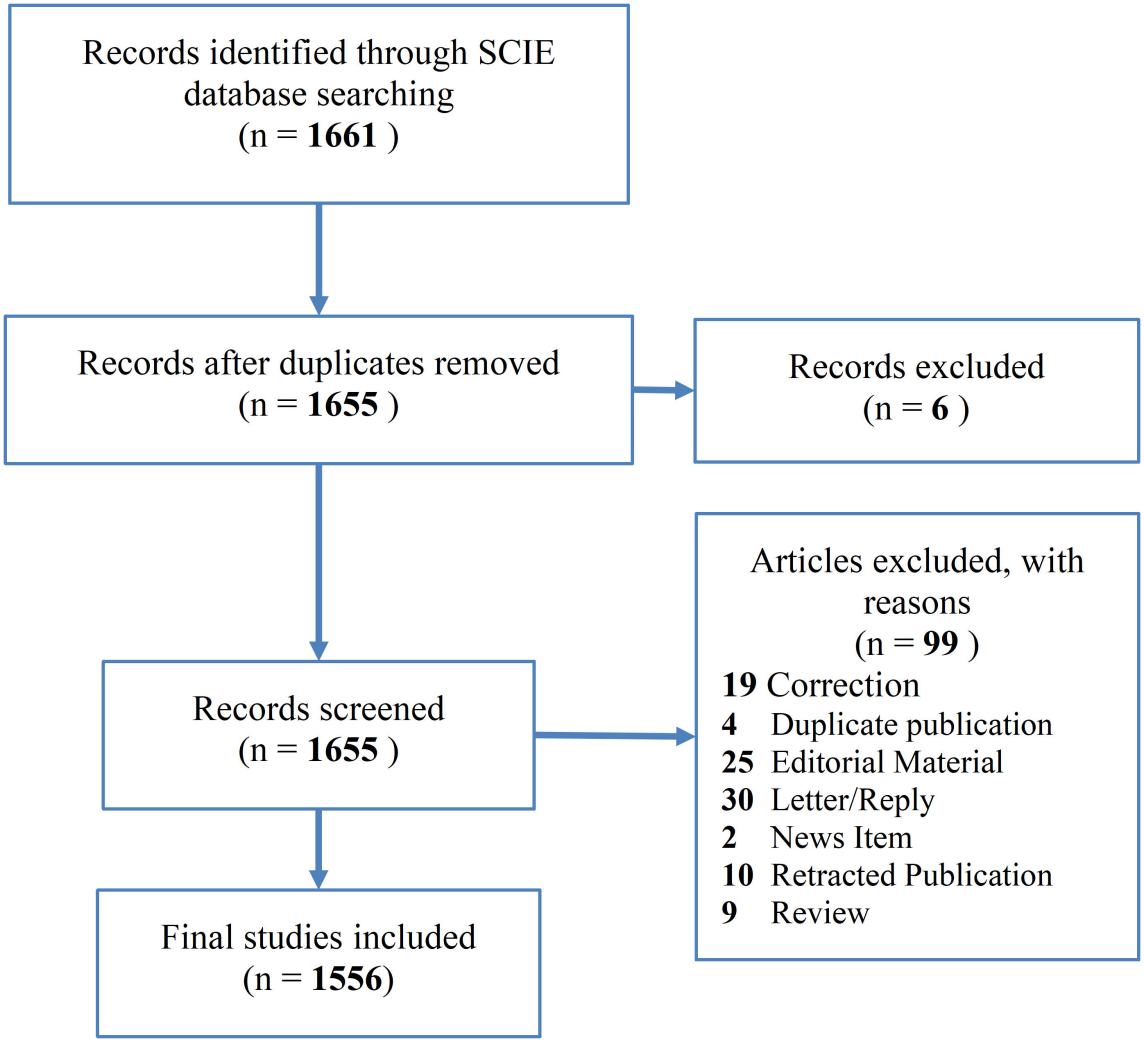
Literature screening process.

This study retrieved a total of 1,661 records from the SCIE database. After deduplication and screening, 1,556 eligible records are ultimately selected for inclusion. **Figure 2** illustrates the flowchart detailing the literature screening process.

### 3.2 Types and annual distribution of publications

**Figure 3:**
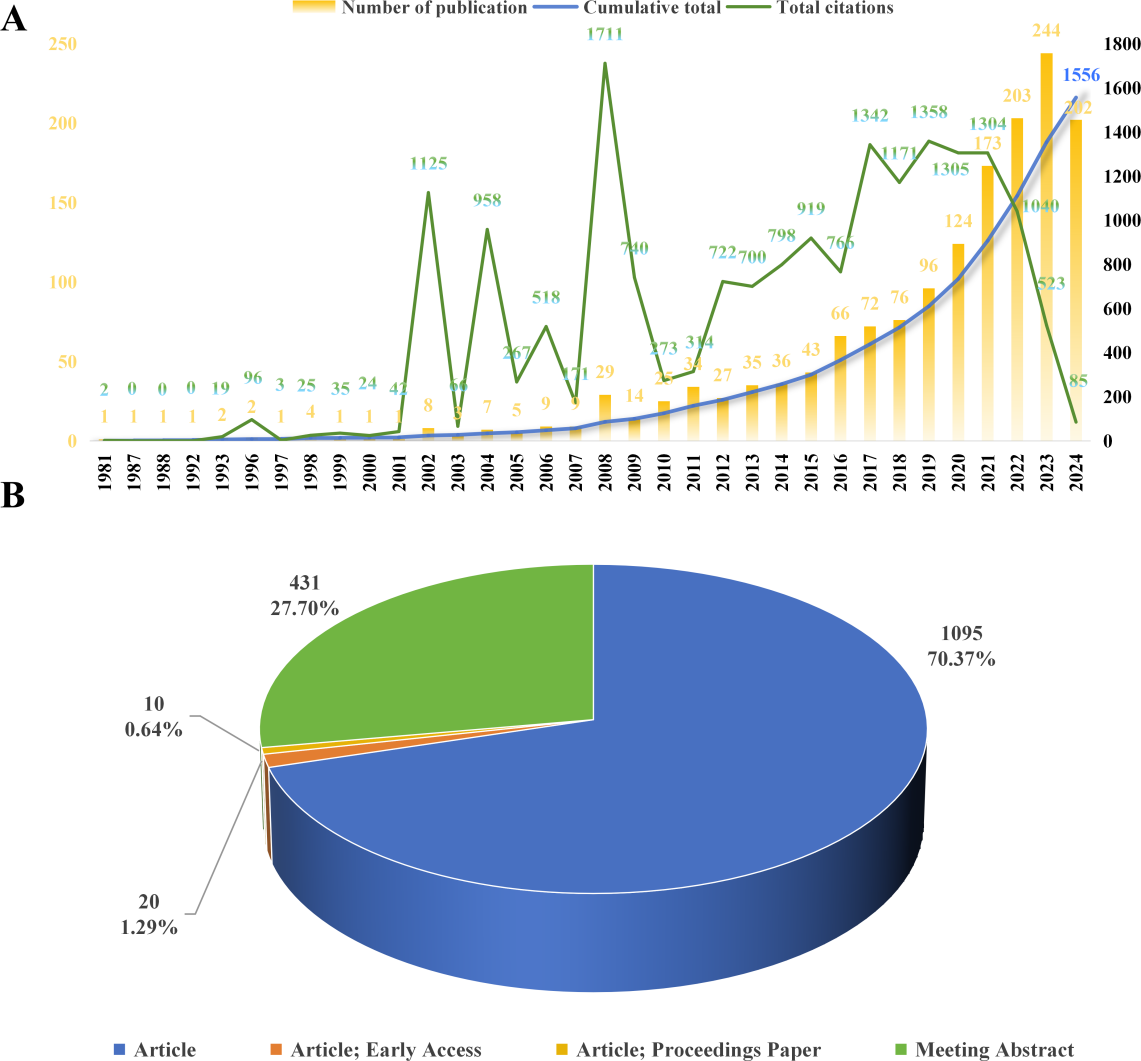
Distribution of publication types and annual publication volume. **(A)** Annual publication volume and citations of publications; **(B)** Distribution of publication types.

A comprehensive review of the publication types and release dates within the field of cancer-related prediction models, spanning from the inception of the SCIE database to 2024, has been conducted (**Figure 3**). During this timeframe, a total of 1,556 relevant publications are released, comprising 1,095 articles (70.37%), 431 meeting abstracts (27.70%), 20 early access articles (1.29%), and 10 proceedings papers (0.64%). The cumulative citation count reaches to 18,422, with an average citation frequency per publication of 11.84. Prior to 2008, only a limited number of publications related to cancer-related prediction models were released annually, suggesting that the field was in its nascent stage. However, from 2008 to 2023 (with 202 publications as of November 15, 2024, which is less than the annual publication volume for 2023), the volume of publications witnesses a significant increase, marking a period of rapid development and maturity for the field. In terms of citation metrics, the average citation frequency per publication for the years 2002, 2004, 2005, 2006, 2008, and 2009 was 50 or higher, with 2002 and 2004 standing out particularly, as the average citation frequency per publication for these years approaches nearly 140. These findings emphasize the growing academic and clinical interest among researchers in cancer-related prediction models.

### 3.3 Countries/regions and institutions

**Figure 4:**
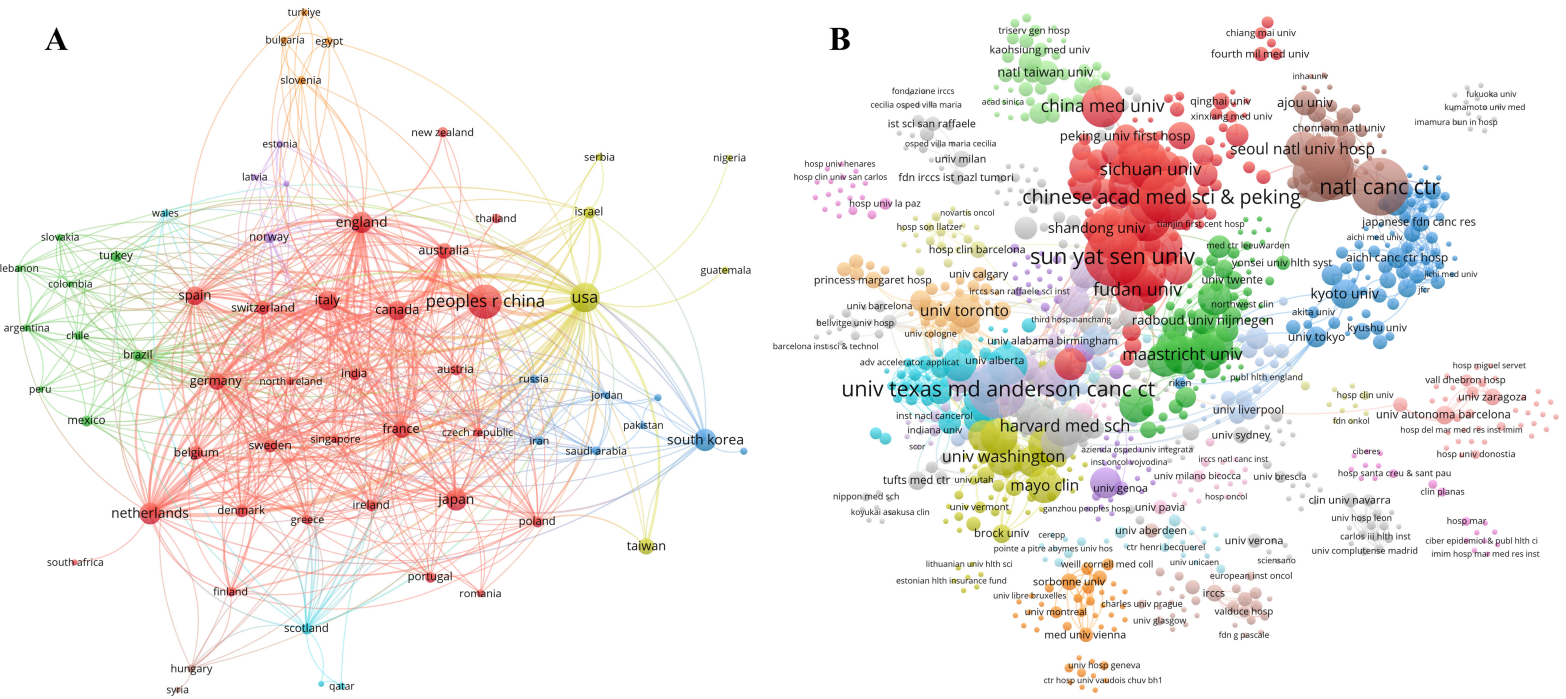
Distribution of countries/regions and institutions. **(A)** A visual mapping of the collaborative networks among countries/regions in relevant publications. Each circle represents a country/region, with the size of the circle proportional to the number of publications; larger circles imply a greater number of publications. **(B)** A visual mapping of the collaborative networks among institutions. Each circle represents an institution, and the size of the circle proportional to the number of publications; larger circles imply a greater number of publications.

The included publications originate from 2,334 institutions across 65 countries/regions, with each contributing at least one relevant publication. Among these countries, China has the highest number of publications (n=625, 40.17%) (**Table 1**). The United States follows in second place, with 346 publications (22.24%). Other countries with significant publication volumes include South Korea (120, 7.71%), the Netherlands (111, 7.13%), Japan (106, 6.81%), England (97, 6.23%), and Italy (78, 5.01%). The collaboration network among these countries/regions is depicted in **Figure 4A**. Specifically, the United States has the closest collaboration ties with other countries/regions, followed by England, the Netherlands, Germany, Italy, France, Canada, and China.

**Table 1:** Top 15 countries/regions ranked by number of publications.

Rank	Country	Publications	Citations	Average citations per publication	Rank in collaboration network strength
1	China (including Taiwan)	625	3801	6.08	8
2	USA	346	6986	20.19	1
3	South Korea	120	1298	10.82	21
4	Netherlands	111	2865	25.81	3
5	Japan	106	913	8.61	11
6	England	97	3300	34.02	2
7	Italy	78	889	11.40	5
8	Canada	67	1680	25.07	7
9	Spain	65	1207	18.57	9
10	France	41	439	10.71	6
11	Germany	41	647	15.78	4
12	Australia	35	405	11.57	12
13	Belgium	22	607	27.59	15
14	Switzerland	21	524	24.95	10
15	Sweden	19	217	11.42	13

The institutions with the highest publication output include Sun Yat-sen University (36 publications, 2.31%), the University of Texas MD Anderson Cancer Center (35, 2.31%), Seoul National University (31, 1.99%), and the Chinese Academy of Medical Sciences & Peking Union Medical College (29, 1.86%). A visual representation of the collaborative networks among these institutions is presented in **Figure 4B**. Notably, the University of Texas MD Anderson Cancer Center exhibits the strongest collaborative ties with other institutions, followed by Seoul National University, Harvard Medical School, the University of California (San Francisco), Erasmus Medical Center, and Sun Yat-sen University (**Table 2**).

**Table 2:** Top 15 institutions ranked by number of publications.

Rank	Organization	Publications	Citations	Average citations per publication	Rank in collaboration network strength
1	Sun Yat-sen University	36	229	6.36	6
2	University of Texas MD Anderson Cancer Center	35	771	22.03	1
3	Seoul National University	31	445	14.35	2
4	Chinese Academy of Medical Sciences & Peking Union Medical College	29	219	7.55	32
5	Nanjing Medical University	29	258	8.90	27
6	Sungkyunkwan University	26	304	11.69	14
7	Peking University	25	101	4.04	25
8	Shanghai Jiao Tong University	24	134	5.58	120
9	Fudan University	23	202	8.78	140
10	China Medical University	21	225	10.71	38
11	Sichuan University	21	234	11.14	164
12	Fujian Medical University	20	102	5.10	222
13	Yonsei University	20	187	9.35	30
14	Capital Medical University	19	120	6.32	72
15	Duke University	19	159	8.37	36

### 3.4 Authors and journals

**Figure 5:**
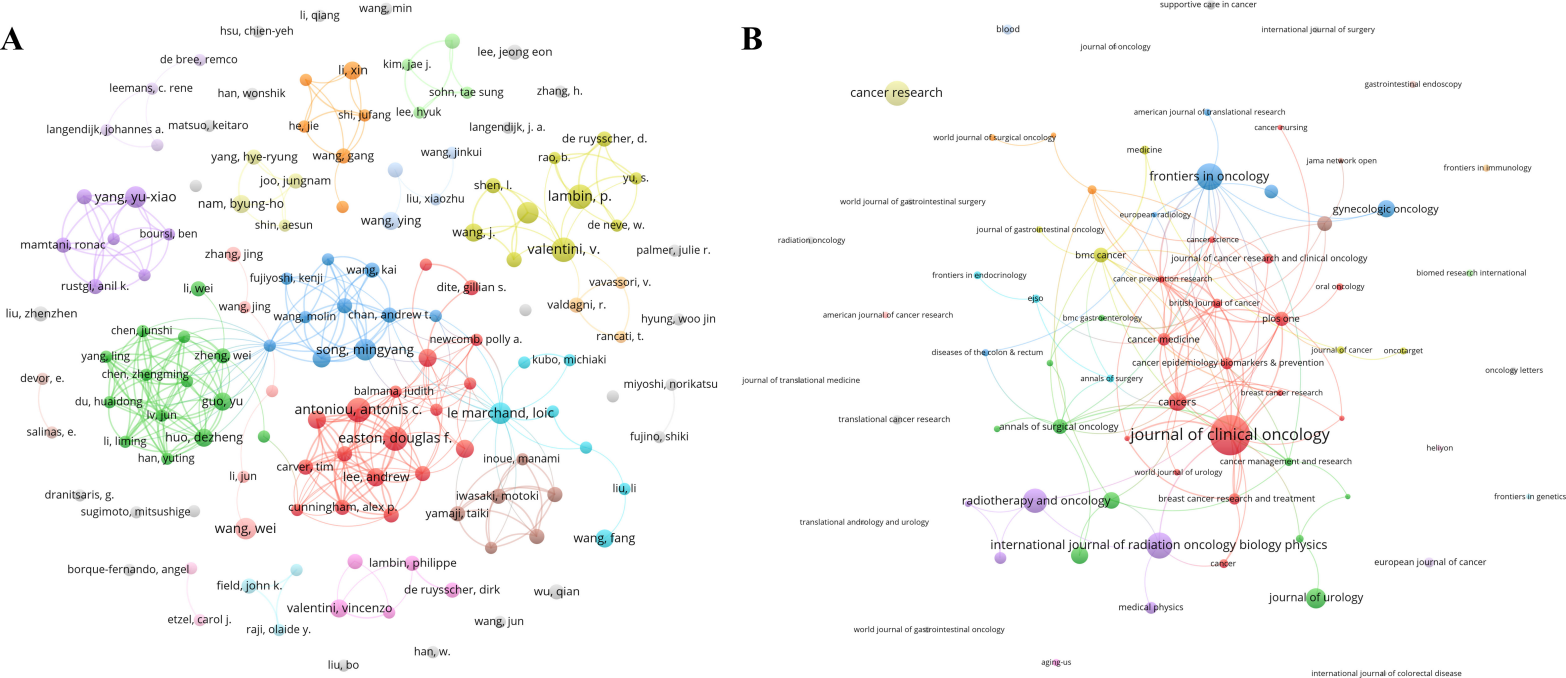
Distribution of authors and journals. **(A)** Visual mapping of the collaboration networks among authors. Each circle represents an author, and a larger circle indicates more publications. **(B)** Visual mapping of the journals. Each circle represents a journal, and a larger circle indicates more publications.

In accordance with Price’s Law ( $=0.749\times \sqrt{8}\approx 2.12$ , authors with three or more publications are designated as core authors. Among the 11,318 authors, 401 are identified as core authors, collectively contributing 1,433 articles (92.10% of the overall publications). A visual representation of authors with four or more publications is depicted in **Figure 5A**. Notably, Antoniou, Antonis C., Easton, Douglas F., Lambin, P., and Valentini, V. emerge as the most prolific authors, each publishing eight articles (**Table 3**). The citations of these authors are 517, 502, 2, and 1, with average citations per publication being 64.63, 62.75, 0.25, and 0.13, respectively.

**Table 3:** Authors with ≥6 publications.

Rank	Author	Publications	Citations	Average citations per publication
1	Antoniou, Antonis C.	8	517	64.63
2	Easton, Douglas F.	8	502	62.75
3	Lambin, P.	8	2	0.25
4	Valentini, V.	8	1	0.13
5	Le Marchand, Loic	7	82	11.71
6	Song, Mingyang	7	6	0.86
7	Wang, Wei	7	49	7.00
8	Yang, Yu-Xiao	7	141	20.14
9	Zhang, Z.	7	5	0.71
10	Guo, Yu	6	29	4.83
11	Hopper, John L.	6	102	17.00
12	Huo, Dezheng	6	25	4.17
13	Lee, Andrew	6	496	82.67
14	Li, Xin	6	22	3.67
15	Mavaddat, Nasim	6	496	82.67
16	Nam, Byung-Ho	6	147	24.50
17	Ogino, Shuji	6	6	1.00
18	Siesling, Sabine	6	57	9.50
19	Valentini, Vincenzo	6	117	19.50
20	Wang, Fang	6	26	4.33
21	Wang, J.	6	5	0.83
22	Wang, Ying	6	12	2.00

Regarding journal distribution, the included publications span 478 journals. Based on Bradford’s Law, the top 36 journals with the highest publication volume are recognized as core journals within the field of cancer-related prediction models. JOURNAL OF CLINICAL ONCOLOGY leads the list with 82 articles (**Table 4**), followed by FRONTIERS IN ONCOLOGY (45 publications) and INTERNATIONAL JOURNAL OF RADIATION ONCOLOGY BIOLOGY PHYSICS (44 publications). A visual mapping of journals publishing five or more is presented in **Figure 5B**. Within the top 36 journals, ANNALS OF ONCOLOGY boasts the highest impact factor (IF) of 56.7. Meanwhile, BRITISH JOURNAL OF CANCER achieves the highest average citation per publication at 61.18, followed by JOURNAL OF UROLOGY with 35.10 (**Table 4**).

**Table 4:** Top 36 journals ranked by publication volume.

Rank	Source	Publications	Citations	Average citations per publication	Impact factor (2023)
1	JOURNAL OF CLINICAL ONCOLOGY	82	994	12.12	42.1
2	FRONTIERS IN ONCOLOGY	45	265	5.89	3.5
3	INTERNATIONAL JOURNAL OF RADIATION ONCOLOGY BIOLOGY PHYSICS	44	431	9.80	6.4
4	CANCER RESEARCH	41	5	0.12	12.5
5	RADIOTHERAPY AND ONCOLOGY	40	447	11.18	4.9
6	JOURNAL OF UROLOGY	31	1088	35.10	6.4
7	CANCERS	27	166	6.15	4.5
8	ANNALS OF ONCOLOGY	25	138	5.52	56.7
9	GYNECOLOGIC ONCOLOGY	25	247	9.88	4.5
10	SCIENTIFIC REPORTS	23	136	5.91	3.8
11	ANNALS OF SURGICAL ONCOLOGY	20	323	16.15	3.4
12	BMC CANCER	20	142	7.10	3.4
13	GASTROENTEROLOGY	20	137	6.85	26.3
14	PLOS ONE	19	389	20.47	2.9
15	INTERNATIONAL JOURNAL OF GYNECOLOGICAL CANCER	18	79	4.39	4.5
16	CANCER MEDICINE	16	134	8.38	2.9
17	CANCER EPIDEMIOLOGY BIOMARKERS & PREVENTION	15	134	8.93	3.7
18	JOURNAL OF THORACIC ONCOLOGY	15	148	9.87	21.1
19	MEDICAL PHYSICS	15	26	1.73	3.2
20	BREAST CANCER RESEARCH AND TREATMENT	14	156	11.14	3.0
21	BLOOD	12	16	1.33	21.1
22	EUROPEAN JOURNAL OF CANCER	12	0	0.00	7.6
23	JOURNAL OF CANCER RESEARCH AND CLINICAL ONCOLOGY	12	36	3.00	2.7
24	BRITISH JOURNAL OF CANCER	11	673	61.18	6.4
25	SUPPORTIVE CARE IN CANCER	11	225	20.45	2.8
26	TRANSLATIONAL CANCER RESEARCH	11	13	1.18	1.5
27	CANCER	10	159	15.90	6.1
28	MEDICINE	10	70	7.00	1.4
29	TRANSLATIONAL LUNG CANCER RESEARCH	10	63	6.30	4.0
30	CANCER MANAGEMENT AND RESEARCH	9	113	12.56	2.5
31	EJSO	9	31	3.44	3.5
32	ANNALS OF SURGERY	8	234	29.25	7.9
33	FRONTIERS IN ENDOCRINOLOGY	8	29	3.63	3.9
34	FRONTIERS IN PUBLIC HEALTH	8	30	3.75	3
35	JOURNAL OF CANCER	8	20	2.50	3.3
36	RADIATION ONCOLOGY	8	33	4.13	3.3

### 3.5 Keywords

#### 3.5.1 Co-occurrence and cluster analysis of keywords

**Figure 6:**
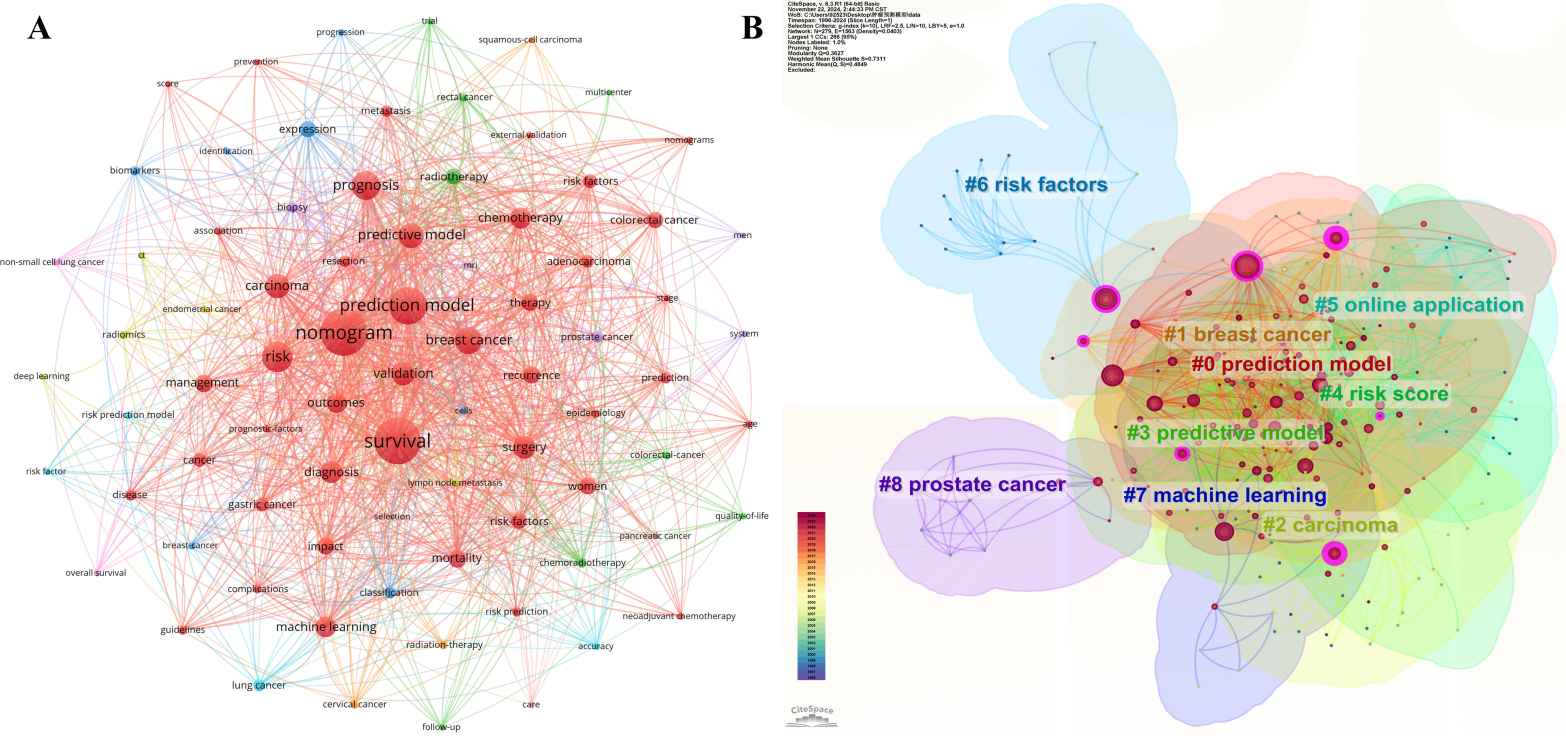
Co-occurrence and cluster of keywords. **(A)** VOSviewer keyword co-occurrence map: Each circle represents a keyword, and a larger circle indicates a higher number of publications associated with that keyword. To ensure readability, only keywords with a frequency of occurrence ≥20 are visually mapped in the VOSviewer keyword co-occurrence map. **(B)** CiteSpace keyword clustering map: Different colored areas represent different clusters of keywords.

In the co-occurrence analysis of keywords, a total of 4,225 keywords are identified. **Table 5** presents the top 30 keywords. With the exception of “prediction model” and “predictive model,” the most frequently occurring keywords include: “nomogram” (192 occurrences), “survival” (191), “risk” (121), “prognosis” (112), “breast cancer” (103), “carcinoma” (93), “validation” (87), “surgery” (85), “diagnosis” (83), “chemotherapy” (80), and “machine learning” (77). These keywords highlight the current primary research directions within the field. The visual representation of the keywords is illustrated in **Figure 6A**.

**Table 5:** Top 30 keywords by frequency of occurrence.

Rank	Keywords	Publications	Citations	Average citations per publication
1	nomogram	192	1864	9.71
2	survival	191	2032	10.64
3	prediction model	151	1463	9.69
4	risk	121	1245	10.29
5	prognosis	112	1119	9.99
6	breast cancer	103	1060	10.29
7	predictive model	96	924	9.63
8	carcinoma	93	972	10.45
9	validation	87	895	10.29
10	surgery	85	854	10.05
11	diagnosis	83	844	10.17
12	chemotherapy	80	884	11.05
13	machine learning	77	677	8.79
14	outcomes	76	787	10.36
15	mortality	66	733	11.11
16	management	65	670	10.31
17	recurrence	63	678	10.76
18	women	63	653	10.37
19	impact	62	668	10.77
20	therapy	62	703	11.34
21	expression	61	690	11.31
22	colorectal cancer	60	585	9.75
23	radiotherapy	57	637	11.18
24	risk-factors	57	597	10.47
25	cancer	51	577	11.31
26	gastric cancer	49	440	8.98
27	risk factors	49	458	9.35
28	adenocarcinoma	46	511	11.11
29	lung cancer	42	370	8.81
30	disease	41	385	9.39

Through further cluster analysis of keywords, a structured outline of the research landscape in this field is presented, enabling researchers and clinicians to grasp a series of knowledge threads that constitute the structure of the field and swiftly comprehend the hotspots within the research area. **Figure 6B** displays the visual mapping of nine keyword clusters, which primarily include “#0 prediction model,” “#1 breast cancer,” “#2 carcinoma,” “#3 predictive model,” “#4 risk score,” “#5 online application,” “#6 risk factors,” “#7 machine learning,” and “#8 prostate cancer”.

#### 3.5.2 Burst term and timeline view analysis

**Figure 7:**
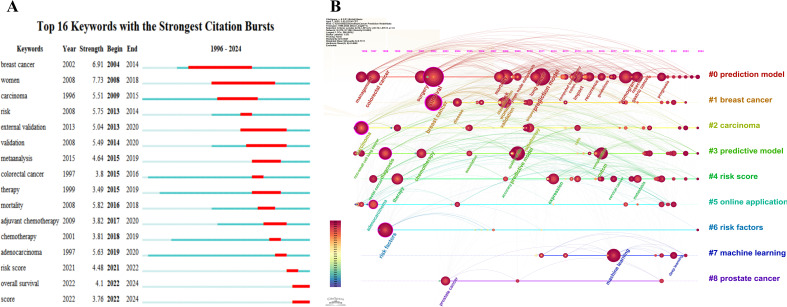
Keyword burst and timeline. **(A)** CiteSpace burst term map: Burst terms typically represent emerging research directions or shifts in field hotspots. The red segment indicates the burst period of the keyword (i.e., the timeframe when its frequency surged abruptly), while the blue segment corresponds to conventional active periods before or after the burst. Strength refers to the Burst Strength — the higher the value, the more rapidly the attention to the keyword has grown. **(B)** CiteSpace timeline map: Temporal analysis of keyword clusters, highlighting longitudinal trends, and pivotal milestones. The horizontal axis represents years, while the vertical axis displays keyword clusters. Keywords within the same-color cluster are thematically related. Connecting lines indicate co-occurrence relationships between keywords, and thicker lines signify stronger associations.

A total of 16 burst terms, each with strengths exceeding 3, are detected. The burst strength of each term is visually displayed in **Figure 7A**, where the length of the red line signifies the duration of the burst. Notably, “breast cancer” was the first burst term to emerge, spanning from 2004 to 2014, with a burst strength of 6.91. The burst terms “breast cancer” and “women” share the longest burst duration, extending from 2008 to 2018. Additionally, “women” exhibits the highest burst strength, with a value of 7.73.

The timeline view analysis provides a profound longitudinal perspective on the evolution of cancer-related prediction models (**Figure 7B**). Clusters such as “#0 prediction model,” “#2 carcinoma,” “#3 predictive model,” “#4 risk score,” and “#6 risk factors” demonstrate sustained vitality, reflecting the enduring interest of the research community. Furthermore, the lifespan of the “#7 machine learning” cluster emphasizes its emerging or continued significance within this field.

#### 3.5.3 Density visualization and timeline view analysis of machine learning

**Figure 8:**
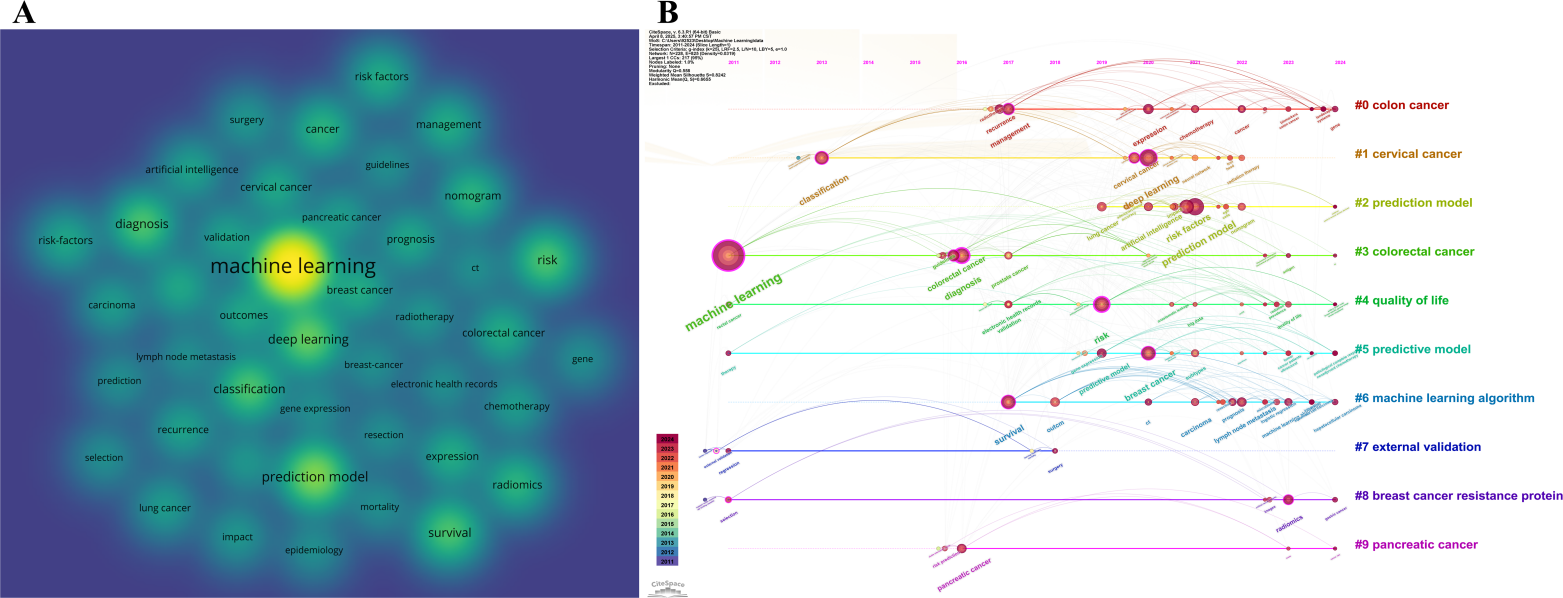
Density visualization and timeline of machine learning. **(A)** VOSviewer keyword density visualization: Each circle represents a keyword, and a brighter circle indicates a higher number of publications associated with that keyword. To ensure readability, only keywords with a frequency of occurrence ≥5 are visually mapped in the density visualization. **(B)** CiteSpace timeline map: Temporal analysis of keyword clusters, highlighting longitudinal trends, and pivotal milestones. The horizontal axis represents years, while the vertical axis displays keyword clusters. Keywords within the same-color cluster are thematically related. Connecting lines indicate co-occurrence relationships between keywords, and thicker lines signify stronger associations.

The dual-perspective visualization reveals the evolving dynamics of machine learning applications in the cancer-related prediction model research (**Figures 8A, B**). **Figure 8A** displays a co-occurrence density map where “machine learning” serves as a central hub, forming an interconnected radiating network with clinical decision nodes (diagnosis and prognosis), technical components (predictive models, risk factors and nomogram), and specific malignancies including breast cancer and colorectal cancer. The timeline network map in **Figure 8B**, organized along a chronological axis (2011-2024) with clustered networks, delineates the evolutionary trajectory of keyword clusters. Notably, prediction and predictive model clusters (#2, #5) demonstrate marked surges in research density following technological breakthroughs in deep learning, artificial intelligence, bagging algorithms, and artificial neural networks. Multidimensional analysis indicates that machine learning applications are progressively extending from predictive models in well-established cancer types (breast cancer, colorectal cancer, and lung cancer) to more complex malignancies such as pancreatic cancer.

## 4 Discussion

To a certain extent, the distribution of publication dates can provide intuitive insights into the pace of development within a particular research field. As illustrated in **Figure 3A**, the annual number of publications on cancer-related prediction models has shown a sharp upward trend since 2015, suggesting that this field has garnered increasing attention and significance from scholars, accompanied by a growing academic and clinical interest. The number of publications by countries/regions and institutions objectively reflects the core research capabilities and influential regions within this research field. China has the highest number of publications (625 publications, 40.17%), followed by the United States (346, 22.24%), South Korea (120, 7.71%), and the Netherlands (111, 7.13%). These countries are prominent scientific contributors and have made substantial contributions to the advancement of cancer-related prediction models. Analysis of the international collaboration networks reveals that the United States, England, and the Netherlands have the closest collaborations with other countries. This close cooperation and the enhancement of international exchanges are conducive to fostering the development of this field, which may be one of the pivotal factors underlying its rapid progression in recent years. By collaborating across borders, researchers can combine their knowledge, skills, and data, leading to more comprehensive and impactful studies. For example, through international collaboration, researchers can access patient populations in different countries, which can improve the generalizability of cancer - related prediction models (21).

Regarding individual contributions, Antoniou, Antonis C., Easton, Douglas F., Lambin, P., and Valentini, V. have published the highest number of publications (eight each), highlighting their significant contributions to the development of cancer-related prediction models. Based on the publications analysis of these authors’ publications, Antoniou, Antonis C., and Easton, Douglas F., from the University of Cambridge, have focused on cancer diagnosis prediction models, including breast cancer risk prediction models (22, 23), epithelial tubo-ovarian cancer risk prediction models (24), and colorectal cancer risk prediction models (25). Meanwhile, Lambin, P. from MAASTRO Clinic, and Valentini, V. from the Università Cattolica del Sacro Cuore, have primarily concentrated on lung cancer prognosis prediction models (26, 27) and colorectal cancer prognosis models (28, 29), respectively.

Frank, I., Weaver, A.L., Cheville, J.C., Blute, M.L., Lohse, C.M., and Zincke, H. received the highest citations (914). In 2002, they developed a scoring system (SSIGN score) based on features such as tumor stage, size, grade and necrosis to predict the prognosis of patients undergoing radical nephrectomy for clear cell renal cell carcinoma (30). Subsequently, Tyrer, J., Duffy, S.W., and Cuzick, J., et al. (895 citations) established a breast cancer prediction model in 2004 that integrates familial and personal risk factors (31) and incorporated it into a computer program to provide personalized risk estimates.

Among the journals, JOURNAL OF CLINICAL ONCOLOGY (82 publications) holds the highest number of publications, followed by FRONTIERS IN ONCOLOGY (45 publications) and INTERNATIONAL JOURNAL OF RADIATION ONCOLOGY (44 publications). Among the top 36 core journals, ANNALS OF ONCOLOGY has the highest impact factor (IF = 56.7), followed by JOURNAL OF CLINICAL ONCOLOGY (IF = 42.1). The journal with the highest average citation per article is BRITISH JOURNAL OF CANCER (61.18 citations), followed by JOURNAL OF UROLOGY (35.10 citations). These journals exert a significant influence on cancer-related prediction models.

### 4.1 Hotspots and future directions

Keywords, serving as pivotal indicators of the content in scholarly publications, provide a crucial tool for identifying research hotspots and developmental trajectories. By conducting a keyword co-occurrence analysis, we can elucidate the relationships among various research topics. This, consequently, offers forward-looking guidance for researchers and clinicians. Among the top-ranked keywords in publications pertaining to cancer-related prediction models, we discerned “nomogram” (frequency = 192), “survival” (191), “risk” (121), “prognosis” (112), “breast cancer” (103), “carcinoma” (93), “validation” (87), “surgery” (85), “diagnosis” (83), “chemotherapy” (80), and “machine learning” (77). Further clustering analysis of these keywords yielded nine clusters, including “#0 prediction model,” “#1 breast cancer,” “#2 carcinoma,” “#3 predictive model,” “#4 risk score,” “#5 online application,” “#6 risk factors,” “#7 machine learning,” and “#8 prostate cancer.” Moreover, in the burst analysis of keywords, we identified a total of 16 burst terms with burst intensities exceeding 3. Recent burst terms include “adjuvant chemotherapy,” “chemotherapy,” “adenocarcinoma,” “risk score,” “overall survival,” and “score.” The results of the keyword co-occurrence, clustering, and burst analyses suggest the following current research hotspots in this field: 1) prediction models for breast cancer (32, 33) and prostate cancer (34, 35); 2) prediction models for cancer prognosis (36), including the prediction of cancer-related complications (37) and responses or adverse reactions subsequent to surgery or chemotherapy in cancer patients (38–40); 3) applications of novel modeling methods, such as machine learning (41, 42); and 4) utilization of tools like risk scores and nomograms in cancer-related prediction models (43–45).

The timeline view analysis further reveals potential future trends in this field, highlighting a notable technological transition in the field of cancer-related prediction models, shifting from traditional risk assessment tools like nomograms and risk scores rooted in conventional statistical models (e.g., logistic regression) toward advanced methodologies such as machine learning and deep learning. The timeline view indicates that the life-cycle of the “#7 machine learning” cluster is exhibiting robust vitality, which reflects a burgeoning interest in machine learning algorithms and artificial intelligence [such as neural networks (46, 47) and deep learning (48, 49)] for enhancing the precision of cancer-related prediction models.

The enormous potential of machine learning and deep learning in biomedicine is increasingly recognized as transformative. As a sophisticated subset of machine learning algorithms, deep learning has been extensively implemented in domains such as image recognition and speech processing. Current and future research priorities in this field primarily focus on two key directions: (1) leveraging deep learning to integrate multimodal data (including radiomics, genomics, and metabolomics) to enhance the predictive accuracy and clinical utility of cancer-related prediction models (50–52); and (2) developing interpretability tools to elucidate model decision-making processes, thereby improving clinician confidence and adoption of machine learning/deep learning-based cancer-related prediction models (53). Emerging applications also demonstrate the feasibility of deep learning in predicting therapeutic efficacy and adverse effects of novel antitumor agents (54–58). For example, Yan, K. et al. (51) developed a dual-channel attention neural network (DANN) that utilizes in-born gene signatures to predict melanoma patients’ responses to immune checkpoint inhibitor therapy. This provides a tool for optimizing therapeutic regimens and minimizing adverse drug reactions.

In recent years, a substantial number of prediction models have been developed in the field of cancer, inevitably leading to multiple models for the same health issue (59). This poses challenges for clinical application selection. Additionally, these prediction model studies may be plagued by issues such as inadequate reporting quality, conflicting conclusions, high risks of bias, and limitations in accuracy and applicability, thereby impeding their clinical use. Systematic reviews may be an important method to select the best model, facilitating the interpretation of the potential applicability and generalizability of prediction models and providing a foundation for further evaluation and validation of models (60, 61). Systematic reviews on cancer-related prediction models may emerge as another research direction in this field (62, 63).

### 4.2 Limitations

This study used bibliometric analysis to provide a multidimensional and comprehensive perspective, as well as quantitative and qualitative insights, into the field of cancer-related prediction models. However, it also has certain limitations: 1) The relevant publications were exclusively sourced from the SCIE database and published in English, excluding publications from other databases and in other languages, which may introduce bias. 2) Searching all fields might retrieve many irrelevant publications. To ensure a high relevance of the retrieved publications to the cancer-related prediction models, we restricted the search to the title field. However, this may exclude some relevant publications that were not identified during the search process. 3) Moreover, challenges in accurately identifying authors due to factors like workplace changes, identical names within the same institution, or typographical errors or spelling discrepancies in names posed difficulties in precisely evaluating author contributions, which was an inherent limitation of bibliometric analysis.

## 5 Conclusion

This bibliometric analysis highlights research hotspots and trends in cancer-related prediction models. In recent years, there has been a substantial increase in the number of publications on cancer-related prediction models, with researchers focusing predominantly on adenocarcinoma diagnostic and prognostic models. Furthermore, the novel modeling techniques, such as machine learning algorithms, particularly deep learning algorithms, is likely to be a pivotal research direction both currently and in the future. Systematic reviews of cancer-related predictive models, which could help clinicians select the optimal model for specific clinical conditions, may emerge as the potential research directions.

## Data Availability

The original contributions presented in the study are included in the article/supplementary material. Further inquiries can be directed to the corresponding author.
